# The Delineation of Internal Organs

**Published:** 1916-10-07

**Authors:** 


### THE DELINEATION OF INTERNAL ORGANS.

### A New Electrical Aid to Diagnosis and Treatment.

The medical sensation of the moment, not only from the outside public's point of view but also from that of the profession of medicine itself, is that provided by an editorial article in the *British Medical Journal* a week ago* on a new method for discovering gross abnormalities in internal organs. This invention is due to a young Scot, who graduated as M.D. at an American University whose degrees are not recognised in Great Britain. He could, therefore, not be given a commission in the R.A.M.C., so enlisted in the ranks, and is now known as Sergeant James Shearer, R.A.M.C. The method by which the internal organs can be delineated is based upon electrical principles, which are described with what is perhaps intentional vagueness in the article in the *British Medical Journal*. It is probable enough that until the end of the war the medico-military authorities may object to the disclosure of what promises to be a valuable aid to diagnosis, and hence to treatment, lest the enemy should profit by it as well as ourselves. However that may be, it is at least true that the processes are described in such a way that no one can possibly learn their details or penetrate their underlying principles from the bare outline of them that alone has been published.

### Dr. Shearer's Accomplishment.

What Dr. Shearer claims to have accomplished is succinctly stated in the *British Medical Journal* as follows. His work enables it to be stated, "without any manual or other examination, whether the more important viscera of a living patient—such as the liver, the kidneys, the spleen, and the brain—are intact so far as their gross anatomy is concerned, while at the same time it supplies evidence of any departure from the normal in the nature of a considerable enlargement or diminution in size, or an effusion of blood, or the presence of a foreign body, or the existence of a tear or cut of the visceral surface." In the pictures of internal organs produced by this method, the outline of the organ delineated is clear and distinct, but no details are visible; irregularities of outline due to gross injuries are shown. The blood-vessels of the brain can be shown, as also any effusion of blood from them; but hitherto it has not been found possible to show the brain substance itself.

That vast strides forward will be made in diagnosis, if this process bears out the expectations that have been formed of it in France and foreshadowed in the account which has now been published, is easy enough to understand; it is more difficult without fuller information to predict how great they will be, or indeed where they will stop. Much depends on the further expansion of the Shearer method, on the elimination of its defects (if it has any), and on the augmentation of its range and reliability. Since all rational treatment is based upon diagnosis, it is reasonable to look forward to immensely enhanced results in therapeutics and in surgery as a result of improved diagnosis. Indeed, it is difficult to assign any bounds to the benefits of this discovery, unless it turns out to be subject to limitations not hitherto disclosed.

### The Choice of a Name.

In the communication already quoted five diagrams are given as instances of the present application of the method, which seems not to have received any name up to the present. The discoverer has, of course, the prior right of assigning to his invention any term he may think fit; but as he has up to the present refrained, so far as we know, from bestowing a name upon it, the adoption of his own name is quite the most natural mark of identification for it, and it is possible that the "Shearer method" may be the name under which it will henceforth be known.

The illustrations given are, as already mentioned, five in number. One is the outline of a brain; or, rather, since the brain substance itself is not shown, of the blood-vessels of a brain, with a large blotch of irregular shape, which represents the site of an effusion of blood. The next is the outline of a kidney from a case of gunshot wound of the back in which injury of the kidney was suspected from the clinical symptoms. The delineation shows an irregular jagged notch in the lower pole of the viscus, a diagnosis which was confirmed by laparotomy and removal of the injured organ. Then there is a delineation of the cæcum and appendix of a patient in whom a diagnosis of recurrent appendicitis was made on the clinical signs and history. The appendix is shown to be markedly thickened, with a little patch in the interior, thought to be possibly a concretion. Unfortunately the surgeon in charge allowed the patient to be transferred to another hospital, and the subsequent history of the case was not followed.

Then follows a case of intestinal injuries. In this the picture was interpreted as showing a wound of the intestine and the presence of a foreign body; the patient died before any operation could be performed, and the correctness of the diagnosis was proved at autopsy. The fifth and last of the series is another somewhat unsatisfactory case; the picture shows the liver of a soldier who had suffered a wound of the axilla, with subsequent tenderness over the lower ribs. In the middle of the liver is seen an irregular lighter patch supposed to represent an abscess. The correctness of this diagnosis is a matter of great interest and practical importance; but, unfortunately, when the report was drawn up no operation had been performed on this patient, and the actual state of his liver was unknown. It would seem on the face of it that a man with a hepatic abscess as large as that purporting to be shown by the Shearer method must have been so ill that immediate exploration would be called for. The fact that no such operation was performed may possibly imply that the man's clinical condition did not seem to call for exploration,

* *British Medical Journal*, September 30, 1916, p. 459.

and this may connote that he had no pus in his liver and that the Shearer diagram is fallacious.

Obviously many more experiments must be made, and some time must elapse, before any definite pronouncement upon this startling novelty in medical research can be profitable. Apart altogether from censorship restrictions, a discreet reserve on so revolutionary a discovery would be the prudent course for the present; and as matters stand it may well be not until after the war that full enlightenment upon the principles involved and their applications in practice will be vouchsafed. In the meanwhile the Army medical authorities can be trusted to give Sergeant Shearer a free hand and ample facilities for continuing his researches, and the upshot must be awaited with hope and patience.

